# Predicting NIH Toolbox Cognition Scores in Older Adults with MCI Using Wearable Sensors: A Machine Learning Approach

**DOI:** 10.1093/geroni/igaf122.2252

**Published:** 2025-12-31

**Authors:** Assma Habadi, Miloš Žefran, Naoko Muramatsu, Lijuan Yin, Woojin Song, Maria Caceres, Elise Hu

**Affiliations:** University of Illinois Chicago, Chicago, Illinois, United States; University of Illinois Chicago, Chicago, Illinois, United States; University of Illinois Chicago, Chicago, Illinois, United States; University of Illinois Chicago, Chicago, Illinois, United States; University of Illinois Chicago, Chicago, Illinois, United States; University of Illinois Chicago, Chicago, Illinois, United States; University of Illinois Chicago, Chicago, Illinois, United States

## Abstract

Early detection of cognitive changes enables timely treatment, intervention, and care planning. However, comprehensive neuropsychological evaluations may not be always feasible. The current study examined the utility of physiological biomarkers for assessing cognitive function among older adults diagnosed with mild cognitive impairment or mild dementia. Participants (N = 23) wore Empatica EmbracePlus, a health-monitoring smartwatch, while completing the NIH Toolbox Cognition Battery (NIHTB-CB). EmbracePlus collected physiological biomarkers continuously throughout the assessment, including blood volume pulse, electrodermal activity, skin temperature, and tri‐axial accelerometry. Machine learning techniques, including supervised learning and ablation analysis, were used to examine correlations between physiological data and NIHTB-CB scores. The analysis showed that tri‐axial accelerometry and electrodermal activity were the leading markers in predicting processing speed tasks scores. For working memory scores, blood volume pulse and temperature were particularly informative. In inhibitory control tasks, both blood volume pulse and electrodermal activity emerged as key markers. Overall, the predicted scores were highly correlated with participants’ actual test scores, achieving correlations as high as 0.88 (Spearman’s rho). Studies with larger samples and additional sensor modalities may further improve predictive accuracy. These findings suggest that health-monitoring smartwatches may serve as a real-time, noninvasive marker of cognition. In turn, such methods can help with timely clinical decision making and offer the possibility of novel clinical interventions.

